# A review on the role of miR-671 in human disorders

**DOI:** 10.3389/fmolb.2022.1077968

**Published:** 2022-12-05

**Authors:** Soudeh Ghafouri-Fard, Arian Askari, Bashdar Mahmud Hussen, Mohammed Fatih Rasul, Sevak Hatamian, Mohammad Taheri, Arda Kiani

**Affiliations:** ^1^ Department of Medical Genetics, School of Medicine, Shahid Beheshti University of Medical Sciences, Tehran, Iran; ^2^ Phytochemistry Research Center, Shahid Beheshti University of Medical Sciences, Tehran, Iran; ^3^ Department of Biomedical Sciences, Cihan University-Erbil, Kurdistan Region, Iraq; ^4^ Department of Pharmacognosy, College of Pharmacy, Hawler Medical University, Erbil, Iraq; ^5^ Department of Pharmaceutical Basic Science, Faculty of Pharmacy, Tishk International University, Erbil, Iraq; ^6^ Department of Anesthesia, Shahid Madani Hospital, School of Medicine, Alborz University of Medical Sciences, Karaj, Iran; ^7^ Urology and Nephrology Research Center, Shahid Beheshti University of Medical Sciences, Tehran, Iran; ^8^ Institute of Human Genetics, Jena University Hospital, Jena, Germany; ^9^ Tracheal Diseases Research Center, National Research Institute of Tuberculosis and Lung Diseases (NRITLD), Shahid Beheshti University of Medical Sciences, Tehran, Iran

**Keywords:** mir-671, cancer, biomarker, expression, prognostic

## Abstract

miR-671 is encoded by a gene on 7q36.1 and contributes to the pathogenesis of a variety of disorders, including diverse types of cancers, atherosclerosis, ischemic stroke, liver fibrosis, osteoarthritis, Parkinson’s disease, rheumatoid arthritis, acute myocardial infarction and Crohn’s disease. In the context of cancer, different studies have revealed opposite roles for this miRNA. In brief, it has been shown to be down-regulated in pancreatic ductal carcinoma, ovarian cancer, gastric cancer, osteosarcoma, esophageal squamous cell carcinoma and myelodysplastic syndromes. Yet, miR-671 has been up-regulated in glioma, colorectal cancer, prostate cancer and hepatocellular carcinoma. Studies in breast, lung and renal cell carcinoma have reported inconsistent results. The current review aims at summarization of the role of miR-671 in these disorders focusing on its target mRNA in each context and dysregulated signaling pathways. We also provide a summary of the role of this miRNA as a prognostic factor in malignancies.

## Introduction

microRNAs (miRNAs) are small-sized non-coding RNAs that partake in the post-transcriptional regulation of gene expression through influencing the stability and translation of transcripts. They are transcribed by RNA polymerase II. The pri-miRNAs produced by this enzyme is capped and polyadenylated. This transcript undergoes a series of cleavage by the Drosha and cytoplasmic Dicer ribonuclease enzymes to produce the stem-loop precursor miRNA and mature miRNA, respectively. The latter is embraced into a RNA-induced silencing complex which can recognize target mRNAs and suppress its translation or destabilize it (Macfarlane and Murphy, 2010). miRNAs participate in the pathoetiology of several disorders through modulation of expression of genes (Hussen et al., 2021), altering signaling pathways (Hussen et al., 2022) or interactions with other types of non-coding RNAs (Ghafouri-Fard et al., 2021a; Taheri et al., 2022).

miR-671 is encoded by a gene on 7q36.1 and involved in the pathogenesis of a range of disorders, including diverse types of cancers, atherosclerosis, ischemic stroke, liver fibrosis, osteoarthritis, Parkinson’s disease, rheumatoid arthritis, acute myocardial infarction and Crohn’s disease. There is not sufficient data about the role of this miRNA in normal physiological processes. However, differential expression of this miRNA in the visceral adipose tissues of patients with non-alcoholic fatty liver disease (Estep et al., 2010) indicates its possible role in metabolic pathways. Moreover, miR-671 has been shown to down-regulate the CDR1 (Cerebellar Degeneration-Related protein 1) gene through an Ago2-slicer-dependent mechanism (Hansen et al., 2011). Moreover, this miRNA has been found to be mainly localized in the nucleus (Hansen et al., 2011). There is no clear evidence about differential expression or functional roles of miR-671-3p versus miR-671-5p. The current review aims at summarization of the role of miR-671 in these disorders focusing on its target mRNA in each context and dysregulated signaling pathways. We also provide a summary of the role of this miRNA as a prognostic factor in malignancies.

### miR-671 in cancers

The influence of miR-671 in the carcinogenesis has been valued by a number of studies in cancer cell lines, animal models of cancers and samples obtained from affected individuals. In the succeeding sections, we define the role of miR-671 in the carcinogenesis based on these three lines of evidence.

## Cell line studies

### Up-regulation of miR-671 in cancer cell lines

**FIGURE 1:**
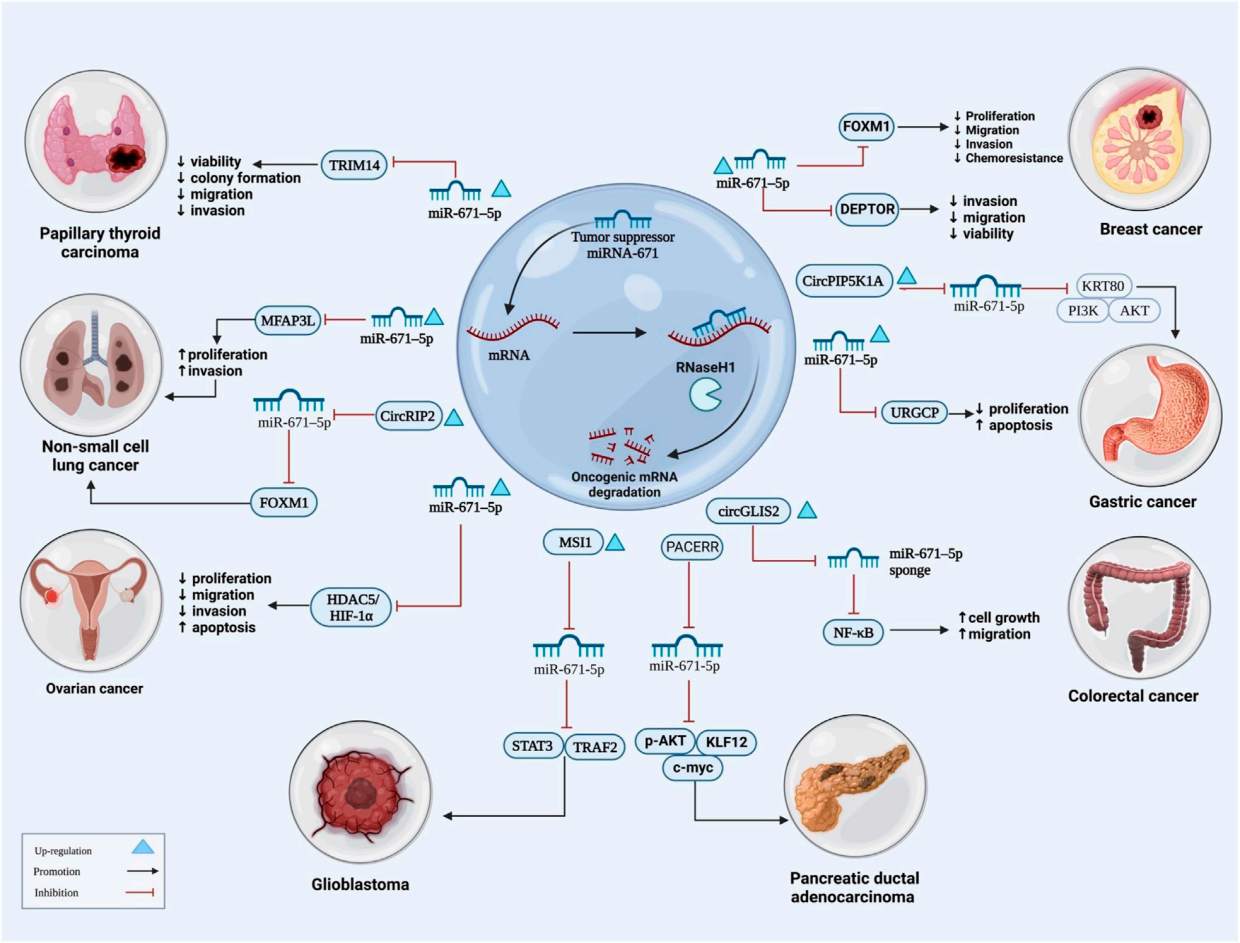
The illustration shows signaling pathways underlying the role of miRNA-671 is as a tumor suppressor miRNA in cancers. miRNA-671 inhibits many signaling pathways and carcinogenic mRNAs, resulting in increased apoptosis while lowering proliferation, migration, and invasion of cancer cells.

Studies in colorectal cancer cell lines have shown down-regulation of circ_PTPRA. Exosomal circ_PTPRA has been shown to induce cell cycle arrest and inhibit proliferation of colorectal cancer cells. In addition, exosomal circ_PTPRA could promote sensitivity of these cells to radiation, resulting in inhibition of colony formation and induction of apoptosis. Mechanistically, circ_PTPRA functions as a sponge for miR-671-5p to increase SMAD4 levels. Taken together, circ_PTPRA inhibits growth and radioresistance of colorectal cancer cells through down-regulation of miR-671-5p levels. Moreover, suppression of miR-671-5p has also blocked growth and radioresistance of these cells through enrichment of expression of SMAD4 (Yang et al., 2022b). Another study in this type of cancer has shown overexpression of a circular RNA, namely circGLIS2. This circRNA is sponged by miR-671. Over-expression of circGLIS2 has led to activation of NF-ƙB pathway and induction of production of pro-inflammatory chemokines leading to stimulation of tumor-associated inflammatory responses *via* recruitment of leukocytes. Taken together, circGLIS2 activates NF-ƙB signaling and promotes migratory ability of colorectal cancer cells through adsorbing miR-671 (Figure 1) (Chen et al., 2020a). Another functional study in colorectal cancer cells has shown the effect of miR-671-5p up-regulation in enhancement of cell proliferation, migratory capacity, and invasiveness of these cells, whereas its downregulation has led to reverse effects. Therefore, miR-671-5p has been suggested as an oncogenic miRNA in colon cancer which exerts its effects through targeting Tripartite Motif Containing 67 (TRIM67) (Jin et al., 2019), a gene, that is, possibly involved in zinc ion binding activity, regulation of protein localization and negative regulation of Ras protein signal transduction (https://www.genecards.org/cgi-bin/carddisp.pl?gene=TRIM67).

miR-671-3p has also been shown to exert oncogenic roles in glioma cells through targeting CKAP4 (Lu et al., 2018). Moreover, it has been demonstrated to be sponged by the tumor suppressor circRNA circDLC1 in these cells (Wu et al., 2022a). A single study in lung cancer cells has shown that miR-671-3p enhances progression of lung cancer through blocking expression of FOXP2 expression in lung cancer (Li et al., 2019b), thus referring to an oncogenic role for this miRNA in lung cancer.

Two independent studies in glioblastoma cell lines have revealed that miR-671-5p has transforming roles. Firstly, more than two-fold upregulated levels of miR-671-5p reduced levels of CDR1-AS/VSNL1 in glioblastoma cell lines A172, CAS-1 and DBTRG. This phenomenon is associated with increased migration and proliferation (Barbagallo et al., 2016). In another study it was demonstrated that if upregulated, miR-671-5p has oncogenic roles, but with competing endogenous features of Circular RNA circ_0001946, this miRNA is suppressed and its suppression is in favor of benign properties (Li and Diao, 2019).

**FIGURE 2:**
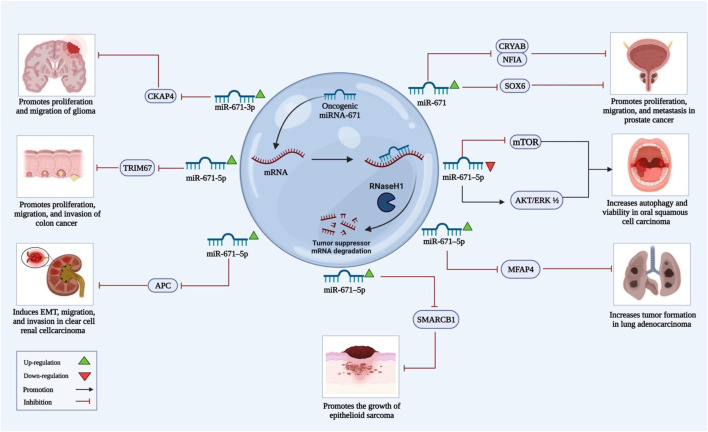
The above illustration shows the roles of miRNA-671, which acts as an oncogene and stimulates cancer growth in several types of cancer. MiRNA-671 can target different tumor suppressor mRNAs and, through inhibition of translation, increase proliferation, migration, and invasion of cancer cells.

Prostate cancer related bioinformatics analysis has shown that miR-671-5p is amongst top differentially expressed miRNAs (Zhu et al., 2020). miR-671-5p has a binding site on the 3′-UTR region of NFIA (Zhu et al., 2020). According to Yang et al., NFIA acts as a tumor suppressor gene in glioma and squamous carcinoma (Yang et al., 2018). Upregulation of miR-671-5p in prostate cancer cell lines reduces NFIA/CRYAB levels and contributes to malignant features like increased proliferation, migration and invasion (Figure 2) (Zhu et al., 2020).

In kidney cancers category, miR-671-5p has been shown to be overexpressed patterns in clear cell renal cell carcinoma (ccRCC) cell lines (786-O and CAKI-1) (Chi et al., 2020). Its overexpression is regulated by HMGA1, which involves in chromatin remodeling (Chiefari et al., 2018). Upregulated levels of miR-671-5p targets APC (a tumor suppressor gene) and gives rise to invasiveness of ccRCC cells (Chi et al., 2020).

### Down-regulation of miR-671 in cancer cell lines

The lncRNA PACERR that sponges miR-671 has been shown to increase the number of M2-polarized cells and enhance proliferation, invasiveness and migration of pancreatic cancer cells. From a mechanistical point of view, PACERR has a role in activation of KLF12/p-AKT/c-myc pathway through sponging miR-671-3p. In fact, this lncRNA is regarded as a regulator of tumor-associated macrophages in pancreatic ductal carcinoma microenvironment (Liu et al., 2022b). Moreover, circ_0092314 has been identified as another non-coding RNA that sponges miR-671 in pancreatic cancer cells, thus increasing expression of S100P and inducing epithelial-mesenchymal transition (EMT) (Shen et al., 2021). These two studies have designated a tumor suppressor effect for miR-671 in pancreatic cancer.

The miR-671-sponging circRNA Circ_0001946 has been shown to be over-expressed in tamoxifen resistant breast cancer cells. This circRNA has been shown to be activated by YY1 in these cells. miR-671-5p mimics could partially reverse the effects of circ_0001946 up-regulation in enhancement of proliferation and invasive properties of drug-resistant breast cancer cells. EGFR has been shown to be the downstream target of miR-671-5p in these cells (Gao et al., 2022). Another study has shown the sponging effect of circSLC8A1 on miR-671 and the impact of this miRNA in the regulation of PTEN/PI3k/AKT pathway (Zhu et al., 2021). Moreover, miR-671-3p has been shown to suppress proliferation and invasiveness of breast cancer cells through modulation of expression of the MTOR-interacting protein DEPTOR (Xia et al., 2020).

Lung cancer cells have also been the subject of functional studies on the role of miR-671. As an example of these studies, Liu et al. (2022a) have shown that the oncogenic role of circRIP2 in this type of cancer is exerted through sequestering miR-671-5p and increasing expression of FOXM1. Moreover, miR-671-5p has been found to inhibit proliferation, migration and invasive aptitude of lung cancer cells through targeting MFAP3L (Ye et al., 2022).

In esophageal squamous cell carcinoma cell lines (including different subtypes of KYSE), elevated levels of FGFR2 activates ERK and AKT signaling pathway and contributes to the malignancy (Li et al., 2019a). Interestingly, miR-671-5p level has shown to be downregulated, hence acting as a tumor suppressor (Li et al., 2019a). Forced expression of this miRNA contributes to diminished levels of FGFR phosphorylation, thus reversing malignant features like proliferation and migration (Li et al., 2019a).

Downregulated levels of miR-671 have also been shown in gastric cancer. In a study conducted by Qiu et al. (2018), reduced level of miR-671-5p has been demonstrated in MKN28 cells compared with normal gastric cells, suggesting an anti-tumor role. Elevating its expression yields decreased ratio of Bcl-2/Bax (increase in BAX), thus promoting apoptosis (Qiu et al., 2018). miR-671-5p targets URGCP and inhibits its expression in MKN28 cells (Qiu et al., 2018). Considering the roles of Up regulator Of Cell Proliferation (URGCP) in the carcinogenesis (Xie et al., 2012; Cai et al., 2015), there is no surprise that targeting it by miR-671-5p has shifted MKN28 cells to normal cell features (Qiu et al., 2018).

Detailed information about the roles of miR-671 in different cancer cell lines is shown in Table 1.

**TABLE 1:** Function of miR-671 in cancer cell lines (Arrows indicate the effects of changes in the expression of mentioned genes (either endogenous or exogenous). ∆: knockdown or downregulation, MPP+: 1-methyl-4-phenylpyridinium).

Tumor type	microRNA type	Levels in cancer cell lines compared with normal cell lines	Interactions	Downstream target of miRNA	Effect of miR-671 up-regulation on its target	Cell line	Associated phenotypes with dysregulation of miR-671	References
Colorectal cancer	miR-671-5p	Upregulated	circ_PTPRA/SMAD4	SMAD4	Inhibition	HCT116 and DLD1	↑ circ_PTPRA: ↓ miR-671-5p ↑ SMAD4: ↓ cell growth ↑ sensitivity to radiation	Yang et al. (2022b)
	miR-671	Downregulated (by GLIS2)	GLIS2/NF-ƙB	NF-ƙB signaling	Inhibition	DLD1, HCT-8, HCT116, RKO, HT-29 and HCT-15	↑ GLIS2: ↓ miR-671-5p ↑ NF-ƙB ↑ migration ↑ motility	Chen et al. (2020a)
	miR-671-5p	Upregulated	TRIM67	TRIM67	Inhibition	SW480, SW620, LOVO, HCT116	↑ miR-671-5p: ↓ TRIM67 ↑ proliferation ↑ migration ↑ invasion	Jin et al. (2019)
Pancreatic ductal adenocarcinoma	miR-671-3p	Downregulated (By PACERR)	PACERR/KLF12/p-AKT/c-myc	KLF12	Inhibition	THP-1 and PATU-8988	↑ PACERR: ↓ miR-671-3p: ↑ KLF12/p-AKT/c-myc ↑ cell invasion ↑ migration	Liu et al. (2022b)
	miR-671	Downregulated	circ_0092314/S100P	SP100P	Inhibition	AsPC-1, BxPC-3, SW-1990 and PaCa-2	↑ circ_0092314: ↓ miR-671: ↑ S100P ↑ EMT ↑ invasion	Shen et al. (2021)
Glioma	miR-671-5p	Upregulated	circDLC1/CTNNBIP1	CTNNBIP1	Inhibition	T98G, LN229, A172, and LN18	↑ circDLC1: ↓ miR-671-5p ↑ CTNNBIP1 ↓ proliferation	Wu et al. (2022a)
	miR-671-3p	Upregulated	CKAP4	CKAP4	Inhibition		↑ miR-671-3p: ↓ CKAP4 ↑ proliferation ↑ migration	Lu et al. (2018)
Breast cancer	miR-671-5p	Downregulated (by Circ_0001946)	Circ_0001946/EGFR	EGFR	Inhibition	MDA-MB-231 and MDA-MB-436	↑ Circ_0001946: ↓ miR-671-5p ↑ EGFR ↑proliferation, ↑ resistance to tamoxifen	Gao et al. (2022)
	miR-671	Upregulated	circSLC8A1/KLF16 PTEN/PI3k/Akt	KLF16	Inhibition	MCF7, T47D, BT474 and MDA-MB-231	↓ circSLC8A1: ↑ miR-671 ↓ PTEN ↑ PI3k/Akt: ↑ proliferation ↑ migration ↑ invasion	Zhu et al. (2021)
	miR-671-3p	Downregulated	DEPTOR	DEPTOR	Inhibition	MCF-7, MDA-MB-231, SK-BR-3	↑ miR-671-3p: ↓ DEPTOR ↓ invasion ↓ migration ↓ viability	Xia et al. (2020)
	miR-671-5p	Downregulated	FOXM1	FOXM1	Inhibition	21T	↑ miR-671-5p: ↓ FOXM1 ↓ proliferation ↓ migration ↓ invasion ↓ chemoresistance	Tan et al. (2019)
	miR-671-3p	Downregulated	HNRNPA2/B1	-	-	MCF-7	↑ HNRNPA2/B1: ↓ miR-671-3p	Klinge et al. (2019)
	miR-671-3p	-	Wnt	-	-	MDA-MB-231	↑ miR-671-3p: ↓ proliferation ↑ apoptosis	Xiong et al. (2018)
	miR-671	Upregulated in drug resistant cell lines	-	-	-	MDA-MB-231	Drug resistant cell lines exhibited up-regulation of miR-671	Chen et al. (2016)
	miR-671-5p	Downregulated	FOXM1	FOXM1	Inhibition	MDA-MB-231, Hs578T, SKBR3, BT-20, MDA-MB-468, MCF-7, and T47D	↑ miR-671-5p: ↓ FOXM1 ↓ proliferation ↓ migration ↓ EMT ↑ sensitivity to chemotherapy	Tan et al. (2016)
Ovarian cancer	miR-671-5p	-	HDAC5/HIF-1α	HDAC5 & HIF-1α	Inhibition	H8910	↑ miR-671-5p: ↓ HDAC5/HIF-1α: ↓ proliferation ↓ migration ↓ invasion ↑ apoptosis	Peng et al. (2022)
Non-small cell lung cancer	miR-671-5p	Downregulated (by CircRIP2)	CircRIP2/FOXM1	FOXM1	Inhibition	A549, H460 and HCC827	↑ CircRIP2: ↓ miR-671-5p ↑ FOXM1 ↑ proliferation ↑ migration	Liu et al. (2022a)
	miR-671-5p	Downregulated	MFAP3L	MFAP3L	Inhibition	H1299, 95D and A549	∆ miR-671-5p: ↑ MFAP3L ↑ proliferation ↑ migration ↑invasion	Ye et al. (2022)
	miR-671-3p	Upregulated	FOXP2	FOXP2	Inhibition	A549 and H1975	∆ miR-671-3p: ↑ FOXP2 ↓ proliferation ↑ apoptosis	Li et al. (2019b)
	miR-671-3p	Downregulated	CCND2	CCND2	Inhibition	A549, H1299, H1650 and H1975	↑ miR-671-3p: ↓ CCND2 ↓ proliferation ↓ invasion	Yao et al. (2019)
Lung adenocarcinoma	miR-671-5p	-	C8orf34-as1/MFAP4	C8orf34-as1 and MFAP4	Inhibition	A549 and H1299	↑ miR-671-5p: ↓ MFAP4 ↑ tumor formation	Han et al. (2021)
Lung squamous carcinoma	miR-671–5p	Downregulated	CDR1as/CDR1	CDR1as	Inhibition	SK-MES-1 and H520	↑ miR-671–5p: ↓ CDR1as ↓ metastasis	Harrison et al. (2020)
Gastric cancer	miR-671-5p	Downregulated	Circ_0000620/MMP2	MMP2	Inhibition	HGC27 and AGS	↑ Circ_0000620: ↓ miR-671-5p ↑ MMP2 ↑ proliferation ↑ invasion	Ren et al. (2021)
	miR-671-5p	Downregulated (by CircPIP5K1A)	CircPIP5K1A/KRT80/PI3K/AKT	KRT80	Inhibition	MKN45, AGS, BGC823, MGC803 and SGC7901	↑ CircPIP5K1A: ↓ miR-671-5p ↑ KRT80/PI3K/AKT ↑ proliferation ↑ Invasion ↑ migration ↑ EMT	Song et al. (2020)
	miR-671-5p	Downregulated	URGCP	URGCP	Inhibition	MKN28	↑ miR-671-5p: ↓ URGCP ↓ proliferation ↑ apoptosis	Qiu et al. (2018)
Glioblastoma	miR-671-5p	Downregulated	MSI1/STAT3/TRAF2	STAT3	Inhibition	Hs683, SW1783, U251, and U87 GBM	↑ MSI1: ↓ miR-671-5p: ↑ STAT3 ↑ TRAF2 ↑ proliferation ↓ radiation sensitivity ↑ cancer stem cell features	Lin et al. (2021)
	miR-671-5p	Upregulated	circ_0001946/CDR1	CDR1	Inhibition	U87 and U251 cells	↑ circ_0001946: ↓ miR-671-5p ↑ CDR1 ↑ apoptosis ↓ migration ↓ invasion	Li and Diao, (2019)
	miR-671-5p	Upregulated	CDR1-AS/CDR1/VSNL1	CDR1-AS and VSNL1	Inhibition	A172, CAS-1, DBTRG, HCT-116, SK-N-BE, SNB-19, U-87 MG	↑ miR-671-5p: ↓ CDR1-AS/VSNL1 ↑ migration ↑ proliferation	Barbagallo et al. (2016)
Papillary thyroid carcinoma	miR-671-5p	-	TRIM14	TRIM14	Inhibition	IHH-4 and TPC-1	↑ miR-671-5p: ↓ TRIM14 ↓ viability ↓ colony formation ↓ migration ↓ invasion	Wang et al. (2021c)
Osteosarcoma	miR-671-5p	Downregulated	SMAD3	SMAD3	Inhibition	hFOB1.19, MG63, U2OS and Saos-2	↑ miR-671-5p: ↓ SMAD3 ↓ EMT ↓ invasion	Hu et al. (2021)
	miR-671-5p	Downregulated	TUFT1	TUFT1	Inhibition	Saos-2, U2OS, and MG-63	↑ miR-671-5p: ↓ TUFT1 ↓ viability ↓ migration ↓ invasion	Ma et al. (2020)
	miR-671-5p	Downregulated	DLEU1/DDX5	DDX5	Inhibition	HOS, MG63, U2OS, and Saos-2	↑ DLEU1: ↓ miR-671-5p ↑ DDX5 ↑ proliferation ↑ migration ↑ invasion	Chen et al. (2019b)
	miR-671-5p	Downregulated	CCND1/CDC34	CCND1/CDC34	Inhibition	U2OS, HOS, Saos-2, MNNG/HOS CI #5, and MG-63	↑ miR-671-5p: ↓ CCND1/CDC34 ↓ proliferation	Xin et al. (2019)
Prostate cancer	miR-671–5p	Upregulated	NFIA/CRYAB	NFIA	Inhibition	RWPE-1, LNCaP, PC-3M, 22RV-1, and C4-2	↑ miR-671–5p: ↓ NFIA/ CRYAB:↑ proliferation ↑ migration ↑ invasion	Zhu et al. (2020)
	miR-671	Upregulated with treatment	SOX6	SOX6	Inhibition	22RV1, DU145, Tsu-Pr1, LNCAP and PC3	↑ miR-671: ↓ SOX6 ↑ proliferation	Yu et al. (2018)
Human cutaneous malignant melanoma	miR-671-5p	Upregulated with treatment	Guizhi Fuling Pills/TPT1-AS1	-	-	A375	Treatment with Guizhi Fuling Pills: ↓ TPT1-AS1 ↑ miR-671-5p ↓ proliferation ↓ migration ↓ invasion	Zhang, (2020)
Clear cell renal cell carcinoma	miR-671-5p	Upregulated	HMGA1/APC	APC	Inhibition	786-O, CAKI-1	↑ HMGA1: ↑ miR-671-5p ↓ APC ↑ migration ↑ invasion ↑ EMT	Chi et al. (2020)
Oral squamous cell carcinoma	miR-671-5p	Downregulated (by CircCDR1)	CircCDR1as/AKT/ERK ½/mTOR	-	-	ca-8113, SCC-15, and HOK	↑ CircCDR1: ↓ miR-671-5p ↓ mTOR ↑ AKT/ERK ½ ↑ autophagy ↑ viability	Gao et al. (2019)
Esophageal squamous cell carcinoma	miR-671-5p	Downregulated	FGFR2/ERK and AKT	FGFR2	Inhibition	KYSE 510, KYSE 410, KYSE 180, KYSE 140, KYSE 30, HKESC1, EC 18, EC 109, EC 9706	↑ miR-671-5p: ↓ ERK and AKT ↓ phosphorylation of FGFR2 ↓ proliferation ↓ Colony formation ↓ migration ↓ invasion ↓ tumorigenesis	Li et al. (2019a)
Hepatocellular carcinoma	miR-671-5p	Downregulated (hypoxia induced)	HIF-1α/TUFT1/Ca2+/PI3K/AKT	TUFT2	Inhibition	Hep3B	Hypoxia: ↓ miR-671-5p: ↑ TUFT2 ↑ Ca2+/PI3K/AKT ↑ proliferation ↑ metastasis	Dou et al. (2019)
Epithelioid sarcoma	miR-671-5p	-	SMARCB1	SMARCB1	Inhibition	HT-1080, Caco-2, and HDFa	↑ miR-671-5p: ↓ SMARCB1	Papp et al. (2014)

### Animal studies

Different animal studies have been performed to evaluate the impact of miR-671 dysregulation on the course of tumor formation. Moreover, a number of other studies have focused on circRNAs that act as molecular sponges for miR-671. For instance, up-regulation of circ_00923 in pancreatic cancer cells has led to down-regulation of miR-671 in tissues of affected animals and enhancement of tumor growth (Shen et al., 2021). On the other hand, over-expression of circ_0001946 has resulted in reduction of glioma growth in animal models (Li and Diao, 2019) Similar to cell line studies, studies in xenograft models of cancers have indicated different results regarding the oncogenic versus tumor suppressor effect of miR-671 (Table 2). For instance, in pancreatic cancer models, down-regulation of miR-671 has been associated with enhancement of tumor growth (Shen et al., 2021). Similar results have been obtained in xenograft models of ovarian cancer (Peng et al., 2022). On the other hand, studies in animal models of colorectal cancer have reported opposite results (Yang et al., 2022b). Detailed information about the role of miR-671 in animal models of cancer is presented in Table 2.

**TABLE 2:** Effect of miR-671 in cancer development based on research in animal models. (∆: knockdown or downregulation).

Tumor type	microRNA type	Animal models	Types of manipulation and engrafted cells	Associated phenotypes with dysregulation of miR-671	References
Pancreatic ductal adenocarcinoma	miR-671	Nude mice	Subcutaneous injection of PaCa-2 cells transfected with specific siRNA against circ_0092314/AsPC-1 cells transfected with circ_0092314 overexpression plasmid	↑ circ_0092314: ↓ miR-671 ↑ tumor growth	Shen et al. (2021)
Glioma	miR-671-5p	BALB/c nude mice	Subcutaneous injection of LN229 cells stably overexpressing METTL3 (lentiviral LV-oe-METTL3)	↑ METTL3: ↓ miR-671-5p ↓ tumor growth	Wu et al. (2022a)
	miR-671-5p	BALB/c nude mice	Subcutaneous injection of U87 cells transfected with circ_0001946	↑ circ_0001946: ↓ miR-671-5p ↓ tumor growth	Li and Diao, (2019)
Glioblastoma	miR-671-5p	BALB/c nude mice	U87MG cells transplantation into the brain which were transfected with miR-671-5p mimics	↑ miR-671-5p: ↓ tumor growth	Lin et al. (2021)
Ovarian cancer	miR-671-5p	BALB/c nude mice	-	∆ miR-671-5p: ↑ tumor growth	Peng et al. (2022)
Colorectal cancer	miR-671-5p	BALB/c nude mice	Subcutaneous injection of vector transfected HCT116 cells with circ_PTPRA	↑ circ_PTPRA ↓ miR-671-5p: ↓ tumor growth ↓ resistance to radiation	Yang et al. (2022b)
Papillary thyroid carcinoma	miR-671-5p	BALB/c nude mice	Subcutaneous injection with TPC-1 cells stably transfected with pMIRNA-miR-671-5p (lentiviral)	↑ miR-671-5p: ↓ tumor growth	Wang et al. (2021c)
Breast cancer	miR-671	BALB/c nude mice	Subcutaneous injection with MCF7 or T47D containing miR-671 inhibiting vectors	↓ miR-671: ↓ tumor growth	Zhu et al. (2021)
Lung squamous carcinoma	miR-671-5p	Athymic nude mice	Intravenous injection with overexpressing miR-671-5p H520 cells	↑ miR-671-5p: ↓ metastasis	Harrison et al. (2020)
Prostate cancer	miR-671-5p	BALB/c nude mice	Subcutaneous injection with PC-3/LV-in-miR-671 (lentiviral)	∆ miR-671-5p: ↓ tumor growth	Zhu et al. (2020)
Clear cell renal cell carcinoma	miR-671-5p	BALB/c nude mice	Intravenous injection with 786-O cells containing miR-671-5p mimics	↑ miR-671-5p: ↑ tumor metastasis	Chi et al. (2020)
Oral squamous cell carcinoma	miR-671-5p	BALB/c nude mice	Subcutaneous injection with Tca-8113 transfected with circCDR1as lentivirus	↑ circCDR1: ↓ miR-671-5p ↑ tumor growth	Gao et al. (2019)
Osteosarcoma	miR-671-5p	BALB/c nude mice	Subcutaneous injection with MNNG/HOS Cl #5 cells (transfected with miR-671-5p)	↑ miR-671-5p: ↓ tumor size	Xin et al. (2019)
Esophageal squamous cell carcinoma	miR-671-5p	BALB/c nude mice	Subcutaneous injection with KYSE180 cells transfected with miR-671-5p mimics	↑ miR-671-5p: ↓ tumor size	Li et al. (2019a)

### Studies in human samples

Expression of miR-671-5p has been increased in colon cancer tissues. Notably, up-regulation of miR-671-5p in this type of cancer has been associated with involvement of lymph nodes, TNM stage, and low overall survival time of affected individuals (Jin et al., 2019). In tumor associated macrophages of pancreatic cancer patients, the lncRNA PACERR that sponges miR-7671 has been shown to be over-expressed in association with poor prognosis of patients (Liu et al., 2022b).

Studies in clinical samples of breast cancer have reported different results regarding the expression of miR-671. First, the miR-671-sponging circRNA circ_0001946 has been shown to be over-expressed in breast cancer tissues, leading to down-regulation of miR-671 (Gao et al., 2022). Although two other studies have reported down-regulation of miR-671-3p (Xiong et al., 2018) and miR-671-5p (Tan et al., 2016) in breast cancer samples, another study has demonstrated up-regulation of miR-671 in another cohort of breast cancer patients (Zhu et al., 2021).

Several studies have shown the impact of miR-671 dysregulation on survival of patients with different kinds of cancer, including ovarian, colorectal and lung cancers as well as osteosarcoma (Table 3). However, a single study in breast cancer patients has reported lack of association between expression levels of miR-671 and median survival of patients (Xiong et al., 2018). Moreover, abnormal expression of miR-671 has been associated with tumor size, TNM stage or metastasis in some kind of cancers, such as colorectal cancer (Jin et al., 2019), lung cancer (Ye et al., 2022) and renal cell carcinoma (Chi et al., 2020). In prostate cancer, up-regulation of miR-671-5p has been associated with higher Gleason score, and BCR status and poor prognosis, but not with tumor stage and lymph node metastasis (Zhu et al., 2020).

**TABLE 3:** Abnormal levels of miR-671 in clinical specimens.

Tumor type	microRNA type	Samples	Expression (tumor *vs*. normal)	Kaplan-Meier and Cox regression analyses (Impact of miR-671 dysregulation)	Association of miR-671 levels with clinicopathologic features	Reference
Pancreatic ductal adenocarcinoma (PDAC)	miR-671-3p	46 PDAC tissues + paired ANT	Downregulated	Upregulation is associated with better prognosis	-	Liu et al. (2022b)
	miR-671	40 PDAC tissues + paired ANT	Downregulated	Downregulation is associated with poor prognosis	-	Shen et al. (2021)
Glioma	miR-671-5p	40 glioma tissues + paired ANT	Upregulated	-	-	Wu et al. (2022a)
	miR-671-3p	8 glioma tissues + paired ANT	Upregulated	-	-	Lu et al. (2018)
Breast cancer (BC)	miR-671-5p	56 BC tissues + paired ANT	Downregulated	-	-	Gao et al. (2022)
	miR-671	77 BC tissues + paired ANT	Upregulated	-	-	Zhu et al. (2021)
	miR-671-3p	38 BC tissues + paired ANT + 11 GEO datasets	Downregulated	Upregulation had no effect on median survival	-	Xiong et al. (2018)
	miR-671-5p	30 IDC tissues + paired ANT	Downregulated	-	-	Tan et al. (2016)
Ovarian cancer (OC)	miR-671-5p	92 OC tissues + paired ANT	Downregulated	Downregulation is associated with poor prognosis	-	Peng et al. (2022)
Colorectal cancer (CRC)	miR-671-5p	25 CRC tissues+ 10 healthy controls	Upregulated	Upregulation is associated with poor prognosis	-	Yang et al. (2022b)
	miR-671-5p	115 CRC tissues + paired ANT	Upregulated	Upregulation is associated with poor prognosis/low O-S	lymph node metastasis and TNM stage	Jin et al. (2019)
	miR-671-5p	38 rectal cancer patients (exposed to capecitabine-oxaliplatin and radiotherapy)	Upregulated in TRG1 patients	-	-	Della Vittoria Scarpati et al. (2012)
Non-small cell lung cancer (NSCLC)	miR-671-5p	30 NSCLC tissues + paired ANT	Downregulated	-	-	Liu et al. (2022a)
	miR-671-5p	56 NSCLC tissues + paired ANT	Downregulated	-	advanced TNM stage and lymph node metastasis	Ye et al. (2022)
	miR-671-3p	43 NSCLC tissues + paired ANT	Downregulated	-	tumor size, TNM stage and metastasis	Yao et al. (2019)
	miR-671-3p	40 NSCLC tissues + paired ANT	Upregulated	-	-	Li et al. (2019b)
Lung adenocarcinoma	miR-671-5p	TCGA database	High mRNA expression-based stemness index is associated with higher miR-671-5p	-	-	Han et al. (2021)
	miR-671-3p	72 lung adenocarcinoma tissues including: 19 EGFR-mutated +17 KRAS-mutated + 16 ALK-rearranged + 20 triple negative cancers	Downregulated in ALK-rearranged cases	-	-	Kim et al. (2017)
Lung squamous cell carcinoma (LUSC)	miR-671	478 LUSC tissues + 45 paired ANT	Upregulated	Upregulation is associated with high O-S	-	Chen et al. (2019a)
Gastric cancer (GC)	miR-671-5p	44 GC tissues + paired ANT	Downregulated	-	-	Ren et al. (2021)
	miR-671-5p	25 GC tissues + paired ANT	Downregulated	-	-	Song et al. (2020)
	miR-671-5p	30 GC tissues + paired ANT	Downregulated	-	-	Qiu et al. (2018)
Osteosarcoma (OS)	miR-671-5p	GSE28423 GSE70414 Datasets	Downregulated	-	-	Hu et al. (2021)
	miR-671-5p	GSE28423 GSE28424 Datasets	Downregulated	Downregulation is associated with poor prognosis	-	Ma et al. (2020)
	miR-671-5p	50 OS Tissues + paired ANT	Downregulated	-	-	Chen et al. (2019b)
	miR-671-5p	20 OS tissues + paired ANT + GSE28425	Downregulated	Downregulation is associated with low O-S	-	Xin et al. (2019)
Prostate cancer (PCa)	miR-671-5p	25 PPCa tissues + 15 MPCa tissues + 13 ANT + GSE21032 GSE21036 GSE21034	Upregulated	Upregulation is associated with higher Gleason score, and BCR status and poor prognosis. miR-671-5p is an independent factor for predicting BCR-free survival	Not associated with tumor stage and lymph node metastasis	Zhu et al. (2020)
	miR-671-3p	66 PCa tissues + 60 healthy controls + 8 controls with atypical lesion	Upregulated in black cases	-	-	McDonald et al. (2018)
	miR-671	8 PCa tissues + paired ANT	Upregulated	-	-	Yu et al. (2018)
	miR-671-5p	GSE21032 dataset	Upregulated	-	-	Sadeghi et al. (2016)
Renal cell carcinoma (RCC)	miR-671-3p	13 lRCC tissues + 15 mRCC	Downregulated in metastatic tissues	-	-	Zhu et al. (2016)
Clear cell renal cell carcinoma (ccRCC)	miR-671-5p	90 ccRCC tissues + paired ANT	Upregulated	Upregulation is associated with poor prognosis/low O-S. miR-671-5p is an independent prognostic factor for O-S	advanced TNM stage	Chi et al. (2020)
	miR-671	TCGA database	Upregulated in Mutant BAP1 tumors	Upregulation is associated with poor prognosis/low O-S	-	Ge et al. (2017)
Chordomas	miR-671-5p	7 chordomas with INI1 loss + 12 normal chordomas + 3 nucleolus pulposus (control)	Downregulation of SMARCB1/INI1 results in upregulation of miR-671-5p	-	-	Malgulwar et al. (2017)
Glioblastoma multiforme (GBM)	miR-671-5p	45 GBM tissues + 3 healthy tissues	Upregulated	-	-	Barbagallo et al. (2016)
Esophageal squamous cell carcinoma (ESCC)	miR-671-3p	56 ESCC tissues + paired ANT	Downregulated	-	-	Warnecke-Eberz et al. (2015)
Epithelioid sarcoma	miR-671-5p	30 epithelioid sarcoma tissues + 2 rhabdoid tumor tissues + 2 SMARCB11 epithelioid sarcoma tissues + 3 epithelioid sarcoma with biallelic-deleted SMARCB1 tissues	Upregulated in epithelioid sarcoma tissues	-	-	Papp et al. (2014)
Hepatocellular carcinoma (HCC)	miR-671-5p	265 HCC patients + 354 CHB patients + 205 healthy controls	Upregulated in HCC patients	-	-	Sun et al. (2013)
Prolactinoma	miR-671-5p	15 prolactinoma patients (5 patients treated with bromocriptine)	Downregulated in treated patients	-	-	Wang et al. (2012)
Myelodysplastic syndromes (MDS)	miR-671-5p	19 MDS tissues + 8 healthy controls	Downregulated	-	-	Borze et al. (2011)

ANT, Adjacent normal tissue; PPCa, Primary localized PCa tissues; MPCa, Metastatic PCa tissues; BCR, Biochemical recurrence; O-S, Overall survival; lRCC, Localized renal cell carcinoma; mRCC: Metastatic renal cell carcinoma; IDC, Invasive ductal carcinoma; CHB, chronic hepatitis B; TRG1, Tumor regression grade 1.

Association between miR-671 variants and risk of soft tissue sarcomas has been assessed in a population of Chinese patients and healthy controls. The results of this study has shown association between miR-671 rs1870238 GC + CC and miR-671 rs2446065 CG + GG genotypes and risk of this type of tumor after adjustment for age and smoking (Zhang et al., 2022a).

## Non-malignant conditions

### Cell line studies

Experiments in ox-LDL-treated HUVECs have shown down-regulation of miR-671-5p and up-regulation of circPTPRA expression. These two transcripts have been shown to interact with each other. While circPTPRA silencing has reversed ox-LDL-induced decrease in viability of HUVECs, miR-671-5p downregulation could abolish this effect. Cumulatively, circPTPRA silencing can protect against ox-LDL-associated HUVECs damage through enhancing expression of miR-671-5p (Luo and Zhou, 2022).

**FIGURE 3:**
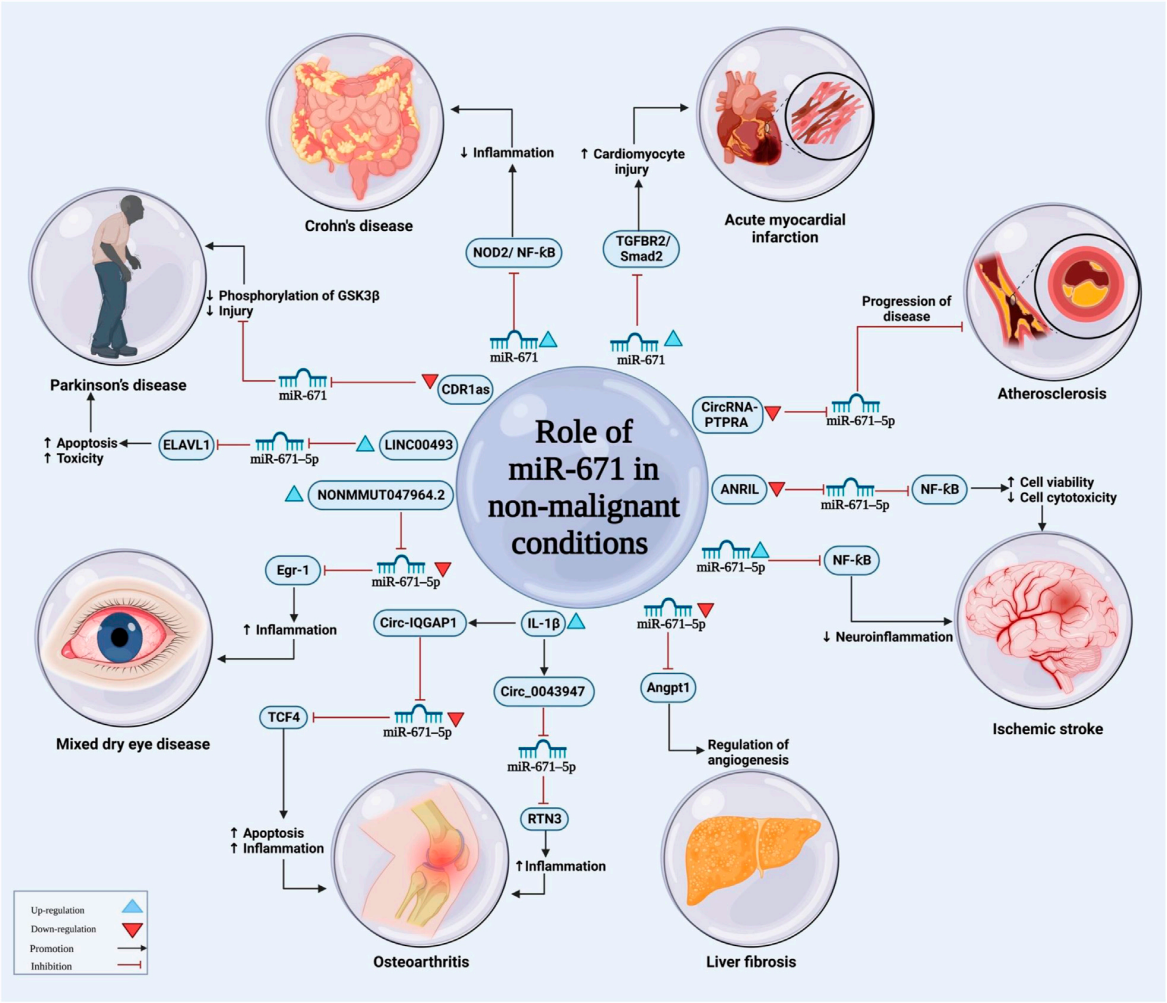
The illustration represents the main functions of miRNA-671 in non-malignant disorders. The level of miRNA-671 expression influences the development of a wide range of disorders by modulation of several signaling pathways.

Another study has shown that the effects of ANRIL silencing in alleviation of neuroinflammatory responses in ischemia is mediated through influencing the miR-671-5p/NF-κB axis (Figure 3) (Deng et al., 2022). Moreover, miR-671-5p could attenuates neuroinflammation through suppression of NF-κB levels (Deng et al., 2021).

miR-671-5p expression has been revealed to be reduced in S1P-induced hepatic stellate cells and TGFβ1-activated hepatic sinusoidal endothelial cells. Moreover, its expression has been negatively correlated with levels of Angpt1 and VWF. Mechanistically, miR-671-5p could target Angpt1 and VWF (Yang et al., 2022b).

miR-671-5p has also been shown to facilitate the effect of lncRNA DLEU1 in the regulation of chondrocytes proliferation, inflammatory responses, and degradation of extracellular matrix (Wu et al., 2022b). Moreover, the sponging effect of circ_0043947 on miR-671-5p is involved in the pathoetiology of IL1β-induced chondrocyte damage and pathogenesis of osteoarthritis (He et al., 2022). Table 4 summarizes the role of miR-671 in the pathogenesis of non-malignant conditions based on the results of cell line studies.

**TABLE 4:** Cell line studies showing the role of miR-671 in non-malignant conditions.

Disease type	microRNA type	Interactions	Cell line	Associated phenotypes with dysregulation of miR-671	Reference
Atherosclerosis	miR-671-5p	CircRNA-PTPRA	HUVECs	ox-LDL treatment: ↑ CircRNA-PTPRA ↓ miR-671-5p progression of disease	Luo and Zhou, (2022)
Ischemic Stroke	miR-671-5p	ANRIL/NF-ƙB	OGD/R HT22	↓ ANRIL: ↑ miR-671-5p ↓ NF-ƙB ↑ cell viability ↓ cell cytotoxicity	Deng et al. (2022)
	miR-671-5p	NF-ƙB	OGD/R HT22	↑ miR-671-5p ↓ NF-ƙB ↓ neuroinflammation	Deng et al. (2021)
Liver fibrosis	miR-671-5p	Angpt1	Primary mouse HSCs	↓miR-671-5p: ↑Angpt1 Regulation of angiogenesis	Yang et al. (2022b)
Osteoarthritis (OA)	miR-671-5p	DLEU1	Human chondrocytes	↑ DLEU1: ↓ miR-671-5p: survival of Chondrocyte, ECM degradation, ↑inflammatory factors	Wu et al. (2022b)
	miR-671-5p	IL-1β/Circ_0043947/RTN3	Human primary chondrocytes	↑ IL-1β: ↑ Circ_0043947 ↓ miR-671-5p ↑ RTN3 ↑inflammation	He et al. (2022)
	miR-671-5p	IL-1β/circ-IQGAP1/TCF4	CHON-001	↑ IL-1β: ↑ circ-IQGAP1 ↓ miR-671-5p ↑ TCF4 ↑ apoptosis ↑ inflammation	Xi et al. (2021)
	miR-671	IL-1β/Circ_0114876/TRAF2	CHON-001	↑ IL-1β: ↑ Circ_0114876 ↓ miR-671 ↑ TRAF2 ↑ inflammation	Wang et al. (2021b)
	miR-671-3p	TRAF3	15 OA+ 15 control Chondrocytes	↑ miR-671-3p: ↓ TRAF3 ↑ matrix ↑ proliferation ↓ inflammation ↓ apoptosis	Liu et al. (2019)
	miR-671	IL-1β	CHON-001	↑ IL-1β and ↑ miR-671: ↓ inflammation ↓ apoptosis	Hou et al. (2019)
Mixed dry eye disease	miR-671-5p	NONMMUT047964.2/Egr-1	MCECs	↑ NONMMUT047964.2: ↓ miR-671-5p ↑ Egr-1 ↑ inflammation	Tang et al. (2022)
Parkinson’s disease	miR-671-5p	LINC00943//ELAVL1	SK-N-SH	MPP + treatment: ↑ LINC00493 ↓ miR-671-5P, ↑ ELAVL1 ↑ apoptosis ↑ toxicity	Zhang et al. (2022b)
	miR-671	CDR1as/GSK3β	PC12	Ex-4 treatment: ↓ CDR1as ↑ miR-671 ↓ phosphorylation of GSK3β ↓ injury	Quan et al. (2021)
Rheumatoid arthritis	miR-671-5p	Circ_0001947/STAT3	RA-FLSs and normal FLSs	↑ Circ_0001947 ↓ miR-671-5p ↑ STAT3 ↑ inflammation ↑ cell migration	Yang et al. (2022a)
	miR-671-5p	Circ-FAM120A/MDM4	RA-FLSs and normal FLSs	Paeoniflorin treatment: ↓ Circ-FAM120A ↑ miR-671-5p ↓ MDM4 ↓ Proliferation ↓ migration ↓ invasion ↓ inflammation	Ghafouri-Fard et al. (2021b)
	miR-671-5p	circ-PTTG1IP/TLR4	RA-FLSs and normal FLSs	∆ circ-PTTG1IP: ↑ miR-671-5p ↓ TLR4 ↑ apoptosis ↓ Proliferation ↓ migration ↓ invasion ↓ inflammation	Chen et al. (2021)
Podocyte injury	miR-671-5p	Wnt/β-Catenin	Human embryonic kidney 293T cells	↑ Wnt/β-Catenin: ↑ miR-671-5p ↑ Injury	Wang et al. (2021a)
Acute myocardial infarction	miR-671	TGFBR2/Smad2	CP-M138, CP-M073	↓ miR-671: ↑ TGFBR2/Smad2 ↑ cardiomyocyte injury	Zhan et al. (2021)
Crohn’s disease	miR-671	NOD2/NF-ƙB	HCT116	↑ miR-671: ↓ NOD2/NF-ƙB ↓ inflammation	Chuang et al. (2014)

ox-LDL, Oxidized low-density lipoprotein; OGD/R, Oxygen glucose deprivation/reperfusion; Ex-4, Exendin-4.

### Animal studies

Expression of miR-671-5p has been down-regulated in the mouse fibrotic liver. Notably, its levels have been negatively correlated with expressions of Angpt1, VWF, sphingosine kinase-1, TGFβ1, HIF1α, HIF2α, and markers of fibrosis. Moreover, expression of miR-671-5p has been lower in hepatic sinusoidal endothelial cells and hepatic stellate cells of CCl4 mice compared with control mice. Administration of miR-671-5p agomir could decrease expressions of Anpgt1 and VWF mRNA and protein levels, and attenuate angiogenesis and fibrosis in the liver of animal models (Yang et al., 2022b). Other investigations in animal models of ischemic stroke, mixed dry eye disease, podocyte injury, acute myocardial infarction and osteoarthritis have verified the role of miR-671 in the pathogenesis of these disorders (Table 5).

**TABLE 5:** Animal studies on the role of miR-671 in non-malignant conditions (MCAO: middle cerebral artery occlusion-reperfusion).

Disease type	microRNA type	Animal models	Results	References
Ischemic Stroke	miR-671-5p	MCAO/R C57/BL6 mice	↓ ANRIL: ↑ miR-671-5p Inhibition of NF-ƙB, Decreased infraction and damaged cells	Deng et al. (2022)
	miR-671-5p	MCAO/R C57/BL6 mice	↑ miR-671-5p: Inhibition of NF-ƙB Decreased inflammation	Deng et al. (2021)
Liver fibrosis	miR-671-5p	Male ICR mice injected with CCL4	↓miR-671-5p: ↑Angpt1 and VWF Induction of angiogenesis in liver fibrosis	Yang et al. (2022b)
Mixed dry eye disease	miR-671-5p	female C57BL/6J mice	↓miR-671-5p: ↑Egr-1 Increased inflammation	Tang et al. (2022)
Podocyte injury	miR-671-5p	BALB/c mice/male CD-1 mice	↑ miR-671-5p: Aggravation of glomerular sclerotic and renal fibrosis	Wang et al. (2021a)
Acute myocardial infarction	miR-671	C57BL/6JNifdc mice	↓ miR-671: ↑ inflammation ↑ apoptosis ↑ fibrosis	Zhan et al. (2021)
Osteoarthritis	miR-671	C57BL/6 male wild-type mice	↑ miR-671: ↓ progression of disease	Hou et al. (2019)

### Studies in human samples

A high throughput sequencing study in pseudoexfoliation syndrome has led to identification of four aberrantly expressed miRNAs among them being miR-671-3p (Tomczyk-Socha et al., 2022). miR-671-5p has also been among miRNAs participating in the pathogenesis of periodontitis through establishment of ceRNA regulatory network regulating autophagy (Bian et al., 2022). miR-671 has also been found to be down-regulated in patients with rheumatoid arthritis (Tang et al., 2019), hand, foot, and mouth disease (Lin et al., 2020), placenta accreta spectrum (Chen et al., 2020b), coronary artery disease (Zhong et al., 2020), Parkinson’s disease (Uwatoko et al., 2019) and Kawasaki disease (Zhang et al., 2018). Table 6 shows the detailed information about the role of this miRNA in human diosrders.

**TABLE 6:** Human studies on the role of miR-671 in non-malignant conditions.

Disease type	microRNA type	Number of clinical samples	Expression (case *vs*. control)	Expression assays	References
Atherosclerosis (AS)	miR-671-5p	30 AS patients + 30 healthy controls	Downregulated	Applied Biosystems 7500 Fast Real-Time PCR system	Luo and Zhou, (2022)
Liver fibrosis	miR-671-5p	20 liver fibrosis patients + 6 healthy controls	Downregulated	ABIPrism 7300 sequence detecting system	Yang et al. (2022b)
Osteoarthritis (OA)	miR-671-5p	30 OA patients + 20 controls	Downregulated	SYBRTM Green kit on ABI7500 PCR System	Wu et al. (2022b)
	miR-671-5p	32 OA patients + 32 controls	Downregulated	SYBR	Xi et al. (2021)
	miR-671	30 OA patients + 20 controls	Downregulated	SYBR Premix ExTaq at ABI Prism 7500	Wang et al. (2021b)
	miR-671-3p	41 early OA patients + 50 late OA patients	Upregulated in early OA patients	Illumina’s NextSeq 550 system	Ali et al. (2020)
	miR-671-3p	15 OA patients + 15 controls	Downregulated	7900 Real-time system	Liu et al. (2019)
	miR-671	20 OA patients + 20 controls	Downregulated	SYBR Green PCR Mix reagent	Hou et al. (2019)
	miR-671-3p	12 OA patients + 12 controls	Downregulated	SurePrint G3 Human miRNA, 8X60K platform (microarray) + ABI 7300 Real-Time PCR System	Ntoumou et al. (2017)
Pseudo exfoliation syndrome (PEX)	miR-671-3p	5 PEX patients + 5 healthy controls	Downregulated	Illumina MiSeq instrument	Tomczyk-Socha et al. (2022)
Periodontitis	miR-671-5p	GSE10334, GSE16134, and GSE54710 datasets (Validation in 5 periodontitis tissues + 5 adjacent healthy tissues)	Upregulated	TB Green Premix Ex Taq™ RR420A/LightCycler 480 System	Bian et al. (2022)
	miR-671-5p	GSE54710 dataset	Upregulated	-	Wang et al. (2019)
Rheumatoid arthritis (RA)	miR-671-5p	29 RA synovial tissues + 29 normal synovial tissues	Downregulated	SYBR Premix DimerEraser	Yang et al. (2022a)
	miR-671	PBMCs of 18 RA patients + PBMCs of 14 healthy controls	Downregulated	Bio-Rad SYBR Green Super mix	Tang et al. (2019)
	miR-671-5p	23 RA synovial tissues + 21 normal synovial tissues	Downregulated	SYBR	Ghafouri-Fard et al. (2021b)
	miR-671-5p	29 RA synovial tissues + 23 normal synovial tissues	Downregulated	SYBR	Chen et al. (2021)
Atrial fibrillation	miR-671-5p	GSE31821, GSE41177, GSE79768, and GSE68475 datasets	Upregulated	-	Xiao et al. (2021)
Hand, foot, and mouth disease (HFMD)	miR-671-5p	GSE85829, GSE94551, GSE52780, and GSE45589 datasets	Downregulated	-	Lin et al. (2020)
	miR-671-5p	5ESHFMD + 5 MHFMD + 5 healthy controls in initial phase/18 ESHFMD + 18 MHFMD + 18 healthy controls in validation phase	Downregulated	Agilent Technologies (microarray)/ABI 7500 Real-Time PCR System	Jia et al. (2014)
Placenta accreta spectrum (PAS)	miR-671-3p	12 PAS patients + 12 healthy pregnant women in the screening phase/41 PAS patients + 41 healthy pregnant women in the training phase/20 PAS patients + 20 PP patients + 20 PE patients and 20 healthy pregnant women in the validation phase	Downregulated	TB Green™ Premix Ex Taq™ II	Chen et al. (2020b)
Coronary artery disease (CAD)	miR-671-3p	80 CAD patients + 20 healthy controls	Downregulated	LightCycler 480 (Roche)	Zhong et al. (2020)
Multiple system atrophy (MSA)	miR-671-5p	31 probable MSA-C patients + 30 probable MSA-P patients + 28 healthy controls	Downregulated in MSA-P patients	3D-Gene^®^ Human miRNA oligo chip (Ver. 17.0), Applied Biosystem^®^ StepOnePlus™ real time PCR system	Uwatoko et al. (2019)
Parkinson’s disease (PD)	miR-671-5p	28 PD patients + 28 healthy controls	Downregulated	3D-Gene^®^ Human miRNA oligo chip (Ver. 17.0), Applied Biosystem^®^ StepOnePlus™ real time PCR system	Uwatoko et al. (2019)
Kawasaki disease	miR-671-5p	GSE60965 dataset	Downregulated	-	Zhang et al. (2018)
Hepatitis B virus (HBV) infection	miR-671-5p	8 immunotolerant + 8 acute viral hepatitis + 16 no fibrosis + 19 early and 14 fibrosis, + 7 healthy controls	Upregulated in advance fibrosis	Agilent´s human miRNA microarray version V16	Singh et al. (2018)
Blood stasis syndrome (BSS)	miR-671-3p	BSS patients including: 10 QDBS patients + 10 QSBS patients + 10 CCBS patients + 10 HABS patients + 40 diabetic patients without BSS	Upregulated in BSS Patients	Hiseq2000 platform and bioinformatics analysis	Chen et al. (2018)
Localized cutaneous leishmaniasis (LCL)	miR-671	12 LCL patients + 7 healthy controls + GSE55664 and GSE63931	miR-671 levels correlate to a better response to treatment	ABI 7500 real-time PCR instrument	Nunes et al. (2018)
Obesity	miR-671-3p	3 obese patients after LAGB	Downregulated	TaqMan low density arrays Human MicroRNA Panel v1.0 (microarray)	Nardelli et al. (2017)
Graft versus host disease (GVHD)	miR-671-3p	19 acute GVHD patients + 38 non-GVHD patients in training phase/21 acute GVHD patients + 33 non-GVHD patients in validation phase	Upregulated in acute GVHD	TaqMan^®^ Human MicroRNA Array A/GeneAmp(Chen et al., 2020b) PCR System 9700	Zhang et al. (2016)
Intrahepatic cholestasis of pregnancy (ICP)	miR-671-3p	10 ICP patients + 10 healthy pregnant women in initial phase/40 ICP patients + 50 healthy pregnant women in validation phase	Upregulated in ICP patients	ViiA7	Ma et al. (2016)
Non-alcoholic fatty liver disease	miR-671-3p	12 non-alcoholic steatohepatitis (NASH) tissues + 12 non-NASH	Downregulated in NASH	TaqMan miRNA Array v 2.0	Estep et al. (2010)

PE, Pre-eclamptic; PP, Placenta previa; MSA-P, Parkinsonian variant; MSA-C, Cerebellar variant; QDBS, Qi-deficiency and blood stasis syndrome; QSBS, Qi-stagnation and blood stasis syndrome; CCBS, Cold-coagulation and blood stasis syndrome; HABS, Heat-accumulation and blood stasis syndrome; LAGB, laparoscopic adjustable gastric banding; MHFMD, Mild HFMD; ESHFMD, Extremely severe HFMD.

Expression levels of miR-671 can be used as diagnostic marker in placenta accreta spectrum, osteoarthritis and hand, foot, and mouth disease (Table 7). The best AUC values have been obtained in extremely severe cases of hand, foot, and mouth disease where mir-671 levels could differentiate this condition from healthy status with AUC value of 1.00 (Jia et al., 2014).

**TABLE 7:** Diagnostic value of miR-671 in diseases (Mild HFMD: MHFMD, extremely severe HFMD: ESHFMD).

Disease type	microRNA type	Samples	Distinguish between	Area under curve	Sensitivity (%)	Specificity (%)	References
Placenta accreta spectrum (PAS)	miR-671-3p	12 PAS patients + 12 healthy pregnant women in the screening phase/41 PAS patients + 41 healthy pregnant women in the training phase/20 PAS patients+20 PP patients+20 PE patients and 20 healthy pregnant	PAS *vs*. healthy pregnant women	0.70	57	76	Chi et al. (2020)
Osteoarthritis (OA)	miR-671-3p	12 OA patients + 12 controls	OA patients and healthy controls	0.87	-	-	Ntoumou et al. (2017)
Hand, foot, and mouth disease (HFMD)	miR-671-5p	18 ESHFMD + 18 MHFMD + 18 healthy controls	MHFMD *vs*. healthy controls	0.79	82	72	Jia et al. (2014)
			ESHFMD *vs*. healthy controls	1.00	100	100	Jia et al. (2014)
			ESHFMD *vs*. MHFMD	0.82	83	78	Jia et al. (2014)

## Discussion

miR-671 is a miRNA with various roles in human disorders. In the context of cancer, different studies have revealed opposite roles for this miRNA. In brief, it has been shown to be down-regulated in pancreatic ductal carcinoma, ovarian cancer, gastric cancer, osteosarcoma, esophageal squamous cell carcinoma and myelodysplastic syndromes. Yet, miR-671 has been up-regulated in glioma, colorectal cancer, prostate cancer and hepatocellular carcinoma. Studies in breast, lung and renal cell carcinoma have reported inconsistent results which cannot be explained by the differences in the roles of miR-671-3p or miR-671-5p. It is possible that this miRNA exert stage- or grade-specific roles in the carcinogenesis.

miR-671 has functional interactions with circ_PTPRA, circ_0092314, circDLC1, circ_0001946, circSLC8A1, circRIP2, circ_0000620, circPIP5K1A and circCDR1as. In fact, these circRNAs act as molecular sponges for miR-671 to influence expression of miR-671 targets. NF-ƙB, EGFR, PTEN/PI3K/AKT, Wnt, HIF-1α, STAT3 and AKT/ERK/mTOR signaling pathways are among those being influenced by dysregulation of miR-671 in different cancers. Moreover, miR-671 has a role in the regulation of EMT in different tissues. This finding is based on functional studies on the role of this miRNA or circRNAs that sponge this miRNA. Thus, miR-671-targetin therapies might affect progression of cancer, invasiveness and metastatic ability of malignant cells.

miR-671 has also been suggested to predict course of cancers originated from different tissues. This speculation is based on the observed associations between dysregulation of this miRNA and survival of patients as well as correlation between its expression levels and clinicopathological data. However, the role of miR-671 as a diagnostic marker for cancers should be investigated in future. Based on the inconsistencies regarding the exact effects of miR-671 in the development and progression of different cancers, it is not expected that miR-671-targetted therapies enter the clinics in near future. More researches are needed to assign a definite role for this miRNA in each type of cancer.

The impact of miR-671 polymorphisms on risk of cancers has only assessed in sarcoma. Similar studies should be conducted to evaluate the association between these polymorphisms and risk of other cancers.

miR-671 has also a fundamental role in the pathophysiology of non-malignant conditions such as atherosclerosis, ischemic stroke, liver fibrosis, osteoarthritis, Parkinson’s disease, rheumatoid arthritis, acute myocardial infarction and Crohn’s disease. Moreover, it has a potential to be used as a diagnostic marker for placenta accreta spectrum, osteoarthritis and hand, foot, and mouth disease. However, dysregulation of miR-671 in malignant and non-malignant disorders originated from a certain tissue complicates the diagnostic application of this miRNA. Meanwhile, contribution of miR-671 to the pathogenesis of both malignant and non-malignant diseases is best explained by the prominent role of this miRNA in the regulation of activity of signaling pathways the control cell proliferation and apoptosis.

Taken together, miR-671 is a miRNA that can affect several target mRNAs and influence activity of signaling pathways that are involved in a variety of human disorders. However, several questions should be answered in order to propose miR-671-targeted therapies as efficient therapies for human disorders.
